# Increased IL-4 mRNA expression and poly-aromatic hydrocarbon concentrations from children with asthma

**DOI:** 10.1186/1471-2431-14-17

**Published:** 2014-01-23

**Authors:** Nasser M Al-Daghri, Sherif Abd-Alrahman, Hossam Draz, Khalid Alkharfy, Abdul Khader Mohammed, Mario S Clerici, Majed S Alokail

**Affiliations:** 1 Biomarkers Research Program, Biochemistry Department, College of Science, King Saud University, PO Box, 2455, 11451 Riyadh, Saudi Arabia; 2 Prince Mutaib Chair for Biomarkers of Osteoporosis, Biochemistry Department, College of Science, King Saud University, 11451 Riyadh, Kingdom of Saudi Arabia; 3 Department of Clinical Pharmacy, College of Pharmacy, King Saud University, 1451 Riyadh, Saudi Arabia; 4 Chair of Immunology, Milano and Fondazione Don C. Gnocchi, IRCCS, University of Milano, Milano, Italy

**Keywords:** IL4, Polycyclic aromatic hydrocarbons, Asthma, Children

## Abstract

**Background:**

Asthma is the most common chronic childhood disease. Imbalance of cytokines released from T helper cells and environmental factors, such as exposure to poly-aromatic hydrocarbon (PAH), play pivotal roles in the pathogenesis of asthma. The aim of this study was to compare the mRNA expression patterns of Interleukin (IL)-4, interferon (IFN)-γ and Acyl Co A long chain 3 (ACSL3) in peripheral blood leukocytes of children with and without asthma. To correlate the obtained mRNA data with serum IL-4, IFN-γ and PAH levels. Further, to determine the effect of *in vivo* exposure to PAH on mRNA expression of IL-4, IFN-γ and ACSL3 genes in a rat model.

**Methods:**

A total of 170 children below 16 years (85 pediatric asthma patients and 85 matched healthy controls) were randomly selected from the Riyadh Cohort, Saudi Arabia. Gene expression analysis was performed using qRTPCR. Serum IL-4, IFN-γ and PAH were measured using LINCOplex (human multiplex immunoassay kit) and HPLC respectively.

**Results:**

IL-4 mRNA expression was significantly increased (P < 0.05) in children with asthma compared to healthy control group whereas no differences were observed for either IFN-γ or ACSL3 mRNA. Similarly, serum IL- 4 and PAHs concentration was significantly higher as well in children with asthma in whom IFN-γ was also significantly lower. Results obtained in rats showed that exposure to the benzopyrene prototype PAH resulted in a 2.6 fold (P < 0.001) increased IL-4 mRNA expression in blood.

**Conclusion:**

Peripheral blood IL-4 mRNA levels, serum concentration of this cytokine are elevated in children with asthma. Also, elevated levels of PAH were observed in children with asthma. Additionally, PAH administration in rodents resulted in an increased IL-4 mRNA which is supposed to partly mediate the inflammatory response noted in asthma.

## Background

Asthma is the most common chronic childhood disease; this disease is characterized by variable airflow obstruction, inflammation of the airways and bronchial hyper-responsiveness, and its prevalence is increasing in Saudi Arabia [1,2]. The overall prevalence of asthma in Saudi children has been described to ranges from 8% to 25% based on studies carried out over the past three decades [3]. The pathogenesis of asthma has been related to an imbalance of cytokines released from T helper (Th) cells, [4] with an increase in Th2 type cytokines (Interleukin (IL) IL-4, IL-5, and IL-13) and a decrease in Th1 cytokines (IFN-γ and IL-2). IL-4 is a key cytokine in the development of allergic inflammation. The elevated serum levels of IL-4, an essential cofactor for immunoglobulin E (IgE) production, and IL-5, responsible for the final differentiation, activation and recruitment of eosinophils, have been found in patients with asthma [5]. IFN-γ down regulates IL-4 production and its levels are thought to be unaltered in asthmatic patients [6]. Fluid recovered by bronchoalveolar lavage (BAL) from asthmatic airways is enriched with IL-4, IL-5, IL-13 and granulocyte-macrophage colony stimulating factor (GM-CSF) but not IFN-γ, indicating the presence of Th2 subclass CD4+ lymphocytes [7]. ACSL3 belongs to the acyl-CoA synthetase long chain (ACSL) family of genes which encodes key enzymes in fatty acid metabolism [8-10]. ACSL3 has been shown to be associated with asthma susceptibility in specific populations [11,12].

The pathogenesis of asthma is suggested to center around immunological and environmental factors. In particular, factors, such as living in high traffic urban areas and exposure to diesel exhaust particles, repeated exposure to polycyclic aromatic hydrocarbons (PAHs) derived largely from incomplete combustion of organic material, such as fossil fuels; coal, wood and tobacco could be associated with early development of asthma-related symptoms [2,13].

Several studies have been reported the expression cytokine mRNA in BAL cells, bronchial mucosa and induced sputum of asthmatic patients [14]. Studies of peripheral blood T cells in adult asthma suggested that the properties of these cells reflected those of cells in the BAL and bronchial mucosa [15]. The use of blood for gene expression studies offers an advantage over other tissues because of easy accessibility and, therefore, of great interest for clinical studies and human experimental research [16]. We focused on the expression of IL-4, IFN-γ and ACSL3 mRNA in peripheral blood mononuclear cells (PBMC) of Saudi children with and without asthma and sought possible associations between cytokines and PAH levels. Further, to analyze the possible effect of *in vivo* exposure to PAH (Benzopyrene) on IL-4, IFN-γ and ACSL3 mRNA expression, we extended our study and tested the effect of chronic exposure to PAH in Wistar albino rats.

## Methods

### Subjects and study protocol

A total of 170 (85 asthmatic, 85 non-asthmatic) Saudi children and adolescents (16 years old and below) participated in this cross-sectional study. The subjects were selected randomly from the national biomarker screening, biomarkers research program, King Saud University (KSU), Riyadh, Kingdom of Saudi Arabia (KSA). Ethical approval was granted by the Ethics Committee of King Saud University, Riyadh, Saudi Arabia. Children with asthma were selected based on established pediatric diagnosis and medications taken. The parent or guardian of each child were asked to sign a consent form and to answer a questionnaire including demographic information, dietary questions, area of residence (e.g. near the factory, high-traffic area, etc.), presence of a smoker at home and other pertinent questions related to asthma. Asthma wheeze was monitored using international study of asthma and allergies in childhood (ISAAC) questionnaire for wheeze assessment.

### Clinical and biochemical measurements

Clinical and anthropometric parameters, including blood pressure, weight, height and hip and waist circumferences were measured following standard procedures. Body mass index (BMI) was calculated as weight/height^2^ (Kg/m^2^). Fasting blood samples were collected and the serum glucose, triglyceride, total and HDL-cholesterol levels were measured by chemistry auto-analyzer (Konelab, Espoo, Finland) and concentrations of LDL-cholesterol were calculated using Friedwald's formula. IL-4 and IFN-γ concentrations were measured using LINCOplex, human multiplex immunoassay kit based on Luminex 100 system platform (Luminex Corporation, Austin, TX, USA) with an intra-assay variability of <10% and inter-assay variation of <15%. All fasting samples fell within the detection range.

### Quantitation of PAH in serum samples

PAH was measured in serum samples using HPLC according to a previously described method [2]. A stock solution of 12 PAHs mixed standard solutions was prepared by dissolving 1 mg from each PAH in 100 ml acetonitrile. The series of PAHs mix standard (0.0, 0.5, 2.5, 5, 10, 50 and 100 ng ml-1) were prepared in acetonitrile for linearity. Calibration curves were generated by plotting peak area versus concentration. Each subject’s sample was analyzed for a suite of 12 PAHs as previously described [17]. Analytical determination was conducted by using liquid-liquid extraction followed by high performance liquid chromatography with fluorescence detector (HPLC-FLD). Standard calibration curve was presented excellent linearity, with good separation and repeatability. The limit of detection (LOD) was defined as the higher value of either the method blank LOD (three times standard deviation of method blank after subtracting the average blank), or the instrument LOD (signal >3 times the signal to noise ratio). The limit of quantification (LOQ) (signal >10 times the signal to noise ratio). The limits of detection were ranged from 1.2 to 4.0 ng ml-1 (0.001 ppm). The lowest possible standard on the calibration curve was accepted as the LOQ. The calibration curve and recovery validation study were all repeated three times (n = 3). Recovery and precision were estimated by using spiked blank matrix, samples were analysed in duplicate at five levels spread equally over the analytical range. The recoveries were calculated from the analytical signal as the ratio between found and expected expressed in %. The rate of recovery for all 12 PAHs were ranged from 86 to 106%.

### Animals and treatment schedule

Adult male Wistar albino rats (8 weeks old, weighing 200 ± 10 g), were obtained from the experimental animal Care Center, College of Pharmacy, KSU, Riyadh, KSA. Animals were housed under controlled environmental conditions (25 ± 2°C and a 12 h light/dark cycle). Animals had free access to food and water ad libitum. The protocol of this study was been approved by Research ethics committee of College of science, KSU, Riyadh, KSA.

A total of 20 rats were divided into two groups of 10 animals each. Group 1 animals (control group) received an intraperitoneal (i.p.) injection of Benzopyrene (10 mg/kg body weight) for 3 weeks. Animals from group II were injected with normal saline for the same period and were used as control. After 3 weeks of treatment, blood was collected from retro-orbital plexus and animals from both the groups were sacrificed by cervical dislocation. Liver and lung tissues were excised and stored in an all protect tissue reagent (QIAGEN, Hilden, Germany) for further analysis.

### RNA extraction and cDNA synthesis

RNA from peripheral blood was extracted using a leukocyte RNA purification kit (Norgen Biotek, Thorold, Canada) according to the manufacturer’s instructions, while on column DNAse treatment was performed using RNase-Free DNase I Kit (NorgenBiotek). RNA concentrations were quantified by measuring the optical density at 260-nm wavelengths using the Nano-drop ND-1000 spectrophotometer. Purity was determined as the 260/280 nm ratio with expected values between 1.8 and 2.0, indicating absence of protein contamination. The reverse-transcription step was conducted on 500 ng of RNA using the quantitect reverse transcription kit (QIAGEN, Hilden, Germany).

### Quantitaive real time PCR (qRTPCR)

All the experiments were performed in 96 well plates with CFX 96 Real-Time PCR detection system (Bio-Rad, CA, USA). mRNA expression of ACSL3, IL4 and IFN-γ genes was normalized to the house keeping gene, beta actin and GAPDH (Primer sequences for each were presented in Table 1). Each sample was analyzed in triplicate alongside with negative control. RT-PCR was used to amplify the target genes using the cDNA according to the manufacturer’s instructions. Briefly, 1 μl of cDNA was used in a final PCR volume of 20 μl, containing 10 μl of ready-mix (KAPA Biosystems) containing SYBR green, Taq polymerase and buffers, 7 μl of water and 2 μl of left and right primers for housekeeping and target genes. PCR cycles were as follows: three minutes at 95°C followed by 40 cycles of fifteen seconds at 95°C, thirty seconds at 60°C and thirty seconds at 72°C and the data were obtained as cycle threshold (Ct) values.

**Table 1:** Summary of primer sequences used PCR amplifications

**Primers for humans**	**Sequences**	**Product size**
GAPDH-F	GAAGGTGAAGGTCGGAGTC	226
GAPDH-R	GAAGATGGTGATGGGATTTC	
IL-4-F	CACA ACTGAGAAGGAAACCTTC TG	253
IL-4-R	CTCTCTCATGATCGTCTT TAGCCT TTC	
IFN-γ-F	GCAGGTCATTCAGATGTAGCGG	181
IFN-γ-F	TGTCTTCCT TGATGGTCTCCACAC	
ACSL3-F	GAGAGTTTGAACCCGATGGA	186
ACSL3-R	TTGGCACAACAAATCCAATG	
**Primers for rats**	**Sequences**	**Product size**
β-actin-F	TTACTGCCCTGGCTCCTA	144
β-actin-R	ACTCATCGTACTCCTGCTTG	
IL-4-F	CCACGGAGAACGAGCTCATC	101
IL-4-R	GAGAACCCCAGACTTGTTCTTCA	
IFN-γ-F	CCCTCTCTGGCTGTTACTGC	149
IFN-γ-R	TTTCGTGTTACCGTCCTTTTG	
ACSL3-F	GATTGGCTACTCTTCACC	223
ACSL3-R	GAAATCTGCTCCATCTTAT	

### Statistical analyses

Statistical analyses were performed using SPSS version 16.0 (Chicago, IL, USA). Data was represented as a means ± standard deviation. All anthropometrics and clinical parameters were compared between groups by one way Analysis of Variance (ANOVA) followed by Bonferroni *post hoc* test. The P values less than or equal to 0.05 were accepted to indicate statistically significant difference. The 2^-ΔΔCt^ method was used to quantify gene expression [18].

## Results

The anthropometric, clinical and biochemical features of the subjects are presented in Table 2. Children with asthma had significantly lower HDL cholesterol levels than the healthy control group.

**Table 2:** Demographics and biochemical characteristics of studied subjects

	**Non-asthmatic**	**Asthmatic**	***p***
N	85	85	
Age	13.2 ± 2.2	13.7 ± 2.3	0.16
BMI (kg/m^2^)	21.2 ± 5.5	21.3 ± 5.9	0.91
Waist (cm)	66.7 ± 15.1	68.2 ± 16.2	0.54
Hips (cm)	82.8 ± 20.0	83.4 ± 19.6	0.84
SAD (cm)	17.6 ± 7.5	17.7 ± 8.0	0.93
Systolic BP (mmHg)	104.3 ± 9.8	105.8 ± 10.1	0.34
Diastolic BP (mmHg)	68.1 ± 7.8	69.1 ± 8.4	0.43
Cholesterol (mmol/l)	4.3 ± 0.88	4.2 ± 0.79	0.45
Triglyceride (mmol/l)	1.1 ± 0.66	1.0 ± 0.41	0.25
LDL cholesterol (mmol/l)	2.8 ± 0.80	2.7 ± 0.67	0.40
HDL cholesterol (mmol/l)	1.05 ± 0.34	0.88 ± 0.31	**0.001**
Fasting blood glucose (mmol/l)	5.2 ± 1.6	5.3 ± 1.7	0.70
Σ POP (ng/ml)*	21.8 (5.9, 309.3)	99.6 (64.4, 237.6)	**<0.001**

**Note:** Data represented by Mean ± standard deviation.*-Data represented by Median (Inter-quartile range). Independent sample t-test was performed for comparing asthmatic and healthy control. Σ POP represents summation of PAH measured (*i.e.* Naphthalene, Anthracene, Fluorene, Phenanthrene, 4H cyclobenta[def]phenanthrene, Pyrene, Flouranthene, 1,2-benzanthracene, Chrysene ,Benzo(e)pyrene, Benzoacephenanthrylene and Benzo(a)pyrene). P-value significant at *p* < 0.05.

### IL-4, IFN-γ and ACSL3 mRNA in PBMC

mRNA levels were analyzed in freshly prepared RNA from blood. Data are shown as the fold difference in mRNA levels measured in samples of children with asthma compared to those from healthy control subjects (considered as referent with fold expression = 1).

**Figure 1:**
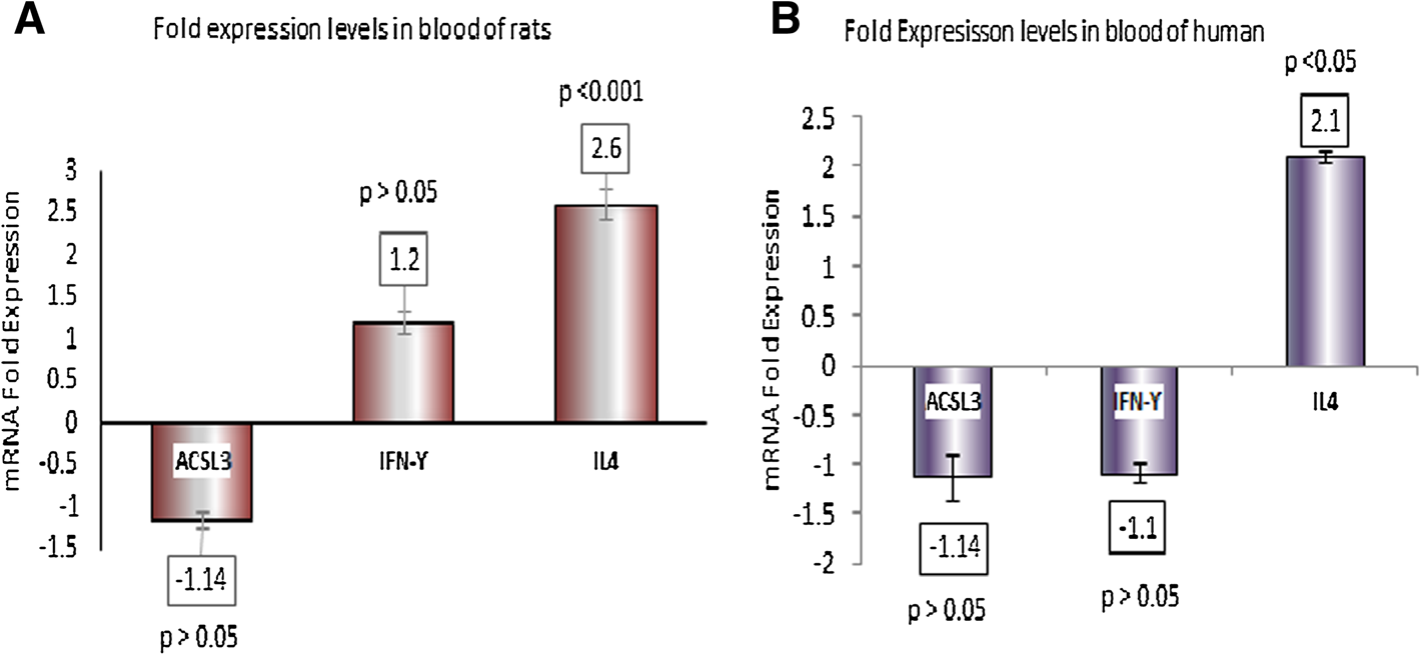
**The expression of ACSL3, IFN-γ and IL-4 mRNA in (A) whole blood from rats treated with benzopyrene and (B) peripheral blood from asthmatic patients. A**: The expression of ACSL3, IFN-γ and IL-4 mRNA in whole blood from rats treated with benzopyrene. Fold difference in ACSL3, IFN-γ and IL-4 transcript levels from treated group compared to transcript level from untreated as referent = 1, as determined by quantitative reverse transcription–polymerase chain reaction. Fold change in mRNA level shown under each bar. Error bars are standard error of the means. **B**: The expression of ACSL3, IFN-γ and IL-4 mRNA in peripheral blood from asthmatic patients. Fold difference in ACSL3, IFN-γ and IL-4 transcript levels from asthmatic patients compared to transcript level from healthy control as referent = 1, as determined by quantitative reverse transcription–polymerase chain reaction. Fold change in mRNA level shown under each bar. Error bars are standard error of the means.

No differences were observed between the asthmatic and healthy control group with respect to IFN-γ and ACSL3 mRNA expression (Figure 1A). In contrast, IL-4 mRNA levels were significantly higher (2.1 fold) (*P* < 0.05) in the asthma compared to the control group (Figure 1B).

### Serum cytokines and PAH levels

**Figure 2:**
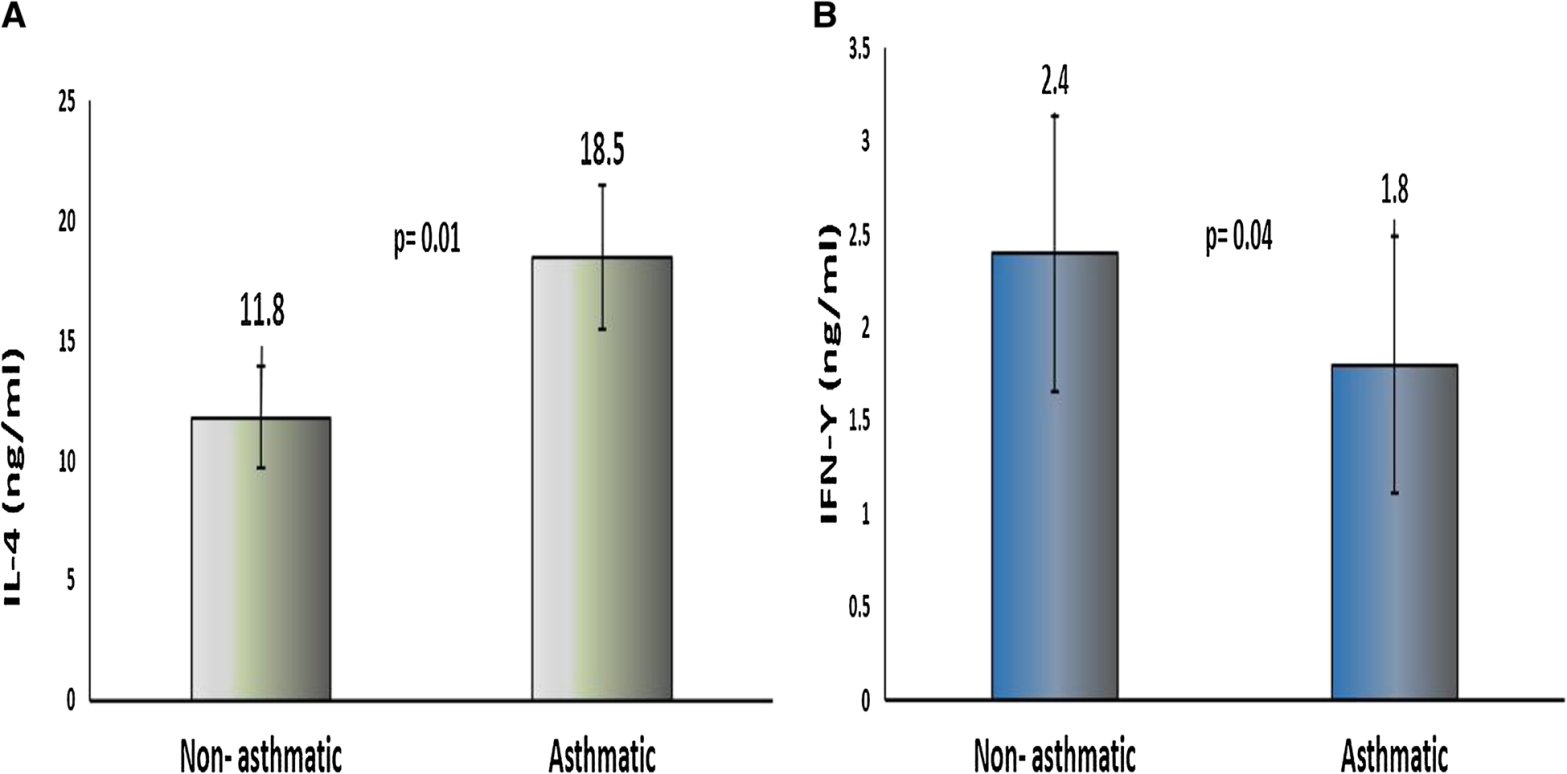
**Comparison of serum levels of IL-4 and IFN-γ in non-asthmatic and asthmatic subjects.** Independent sample t-test was performed for comparing non-asthmatic and asthmatic subjects. Data represented by Mean± standard deviation. Error bars represents standard error of the means. **(A)** Serum IL-4 (ng/ml) concentration among non-asthmatic and asthmatic subjects. **(B)** Serum IFN-γ (ng/ml) concentration among non-asthmatic and asthmatic subjects.

Serum levels of IL-4 and IFN-γ in the two groups analyzed are summarized in Figure 2. Serum IL- 4 levels were significantly higher in children with asthma than in healthy controls [(26.2 ± 2.6 versus 18.2 ± 1.3 ng/ml) (p = 0.01)]. In contrast, serum levels of IFN-γ were significantly lower in children with asthma than in healthy controls [(1.8 ± 0.69 versus 2.4 ± 0.74 ng/ml)] (p = 0.04).

Measurement of PAHs, including Naphthalene, Anthracene, Fluorene, Phenanthrene, 4H cyclobenta[def]phenanthrene, Pyrene, Fluoranthene, 1,2-benzanthracene,chrysene, benzo(e)pyrene, Benzoacephenanthrylene and Benzo(a)pyrene was performed next. Σ POP was calculated by summing up all the above parameters. Mean serum level of PAH was significantly increased in asthma group compared to control individuals [98.8 (63.4, 237.6) versus 22.9 (6.0, 309.3) ng/ml] (p < 0.001) (Table 2).

### Relative expression of IL-4 and IFN- γ related with PAHs

**Figure 3:**
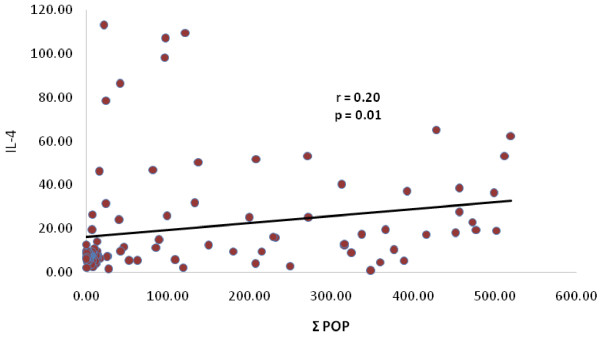
Correlation between serum IL-4 concentrations and PAH levels in studied subjects.

Multivariate analysis using linear regression model showed that IL4 and IFN-γ gene expression levels were significantly correlated with serum PAH levels (Figure 3). Correlation remained significant even after controlling for age or the BMI (Table 3).

**Table 3:** Multivariate analysis using linear regression model to show the association between cytokine IL4 and IFN-γ and gene expression levels with serum PAHs (ΣPOPs) controlled for age and BMI

**Dependent variable**	**Parameters**	**R**^**2**^	**β (standardized coefficients)**	**P value**
IL-4 Gene expression Levels	ΣPOP	0.19	0.36	**<0.001**
	Age		0.18	0.08
	BMI		0.20	**0.05**
IFN-γ Gene expression Levels	ΣPOP	0.26	-0.40	**<0.001**
	Age		0.19	0.06
	BMI		0.31	**0.002**

**Note:** P-value significant at *p* < 0.05.

### Chronic exposure to PAH in the animal model

Compared to the untreated group the treated group of rats had significantly increased IL-4 mRNA expression by 2.6 fold (*P* < 0.001) (Figure 1A). However, no differences were observed between the treated and untreated group of rats with respect to IFN-γ and ACSL3 mRNA expression.

## Discussion

In this study, we investigated the expression of IL-4, IFN-γ and ACSL3 mRNA in PBMCs of the children with and without asthma and speculated that cytokine expression in PBMCs may provide useful information regarding the allergic disease. The results of the present study indicate that leukocyte IL-4 mRNA is over expressed in children with asthma. Also, serum PAH and IL-4 levels were raised in asthmatic condition. We have also demonstrated a significant correlation between cytokine mRNA expression and serum PAH levels. Moreover, *In vivo* chronic exposure of rats to benzopyrene resulted in over expression of IL-4, the pattern that coincided the one observed in asthma group.

The children with asthma had significantly lower HDL cholesterol levels compared to those without asthma. Low serum HDL cholesterol levels are one of the fundamental features of metabolic syndrome and have been shown to affect cholesterol transportation and markers of systemic inflammation in children [19]. The synthesis of the alveolar surfactant is promoted by HDL particles [20], which suggest that HDL may modulate the development of inflammation in the lung. Indeed, several studies have reported that high levels of plasma HDL cholesterol are associated with a decreased risk of allergic diseases [21,22]. A number of studies have found high HDL cholesterol levels to be positively associated with asthma [23]. Whereas, few others found no associations of serum HDL cholesterol with asthma outcomes [17,24]. The results from our report are in agreement with two others from the Third National Health and Nutrition Examination Survey (NHANES) where they found that high serum HDL cholesterol levels to be associated with higher lung function and reduced risk for allergic sensitization in children [21,25]. Whereas, Yiallouros et al. have demonstrated that Low-serum HDL cholesterol in childhood is associated with an increased risk for asthma in adolescence [23].

In asthma, there seems to be an increased expression of Th2 cytokines, with an elevated production of IL-4, IL-5 and IL-13 [5,26]. The present results corroborate with these findings showing increased serum IL-4 expression in children with asthma. These increased serum IL-4 expression was comparable with increased IL-4 mRNA expression in circulating PBMCs from children with asthma. Olivenstein et. Al (14) have shown an increased number of eosinophils and IL-4 and IL-5 mRNA positive cells in the cellular component of induced sputum of asthmatic subjects [14]. Further, Prieto et. al [6] have reported increased IL-4 mRNA expression after cumulative allergen provocation in BAL fluid cells and peripheral CD4^+^ and CD8^+^ T cells. However, these patterns of mRNA levels do not directly reflect protein production and/or secretion by blood cells; it is still not clear how much this accounts for their levels in the circulating blood.

In the current study, finding of lower serum levels of IFN-γ in children with asthma is consistent with earlier study [5]. But, IFN-γ mRNA expression levels in PBMC were not significantly lower in children with asthma than in controls. However, there is conflicting evidence from previous studies observed in humans regarding the relationship between the levels of IFN-γ and asthma. Cho et al. [27] have reported that frequencies of unstimulated airway CD4^+^ and CD8^+^ T cells spontaneously producing IFN-γ were increased in subjects with asthma compared with control subjects [27]. Truyen et al. [28] have reported that increased IFN‒γ mRNA levels were found in the sputum of asthmatic subjects, predominantly in those with moderate to severe asthma [28]. Whereas Kim et al. [29] reported that there is no difference in the IFN-γ level in BAL fluid amongst the children with acute asthma compare to those of no sign of asthma [29]. In children with atopic asthma, Gemou-Engesaeth et al. [30] have detected a high percentage of PBMCs expressing mRNA for IL-4 but a normal percentage for IFN-γ compared with that of control subjects [30].

The data herein suggests that serum PAHs levels were significantly higher in children with asthma than in controls and a significant correlation was observed between expression of IL-4, IFN- γ mRNA and serum PAH levels. The PAH levels detected, likely reflect differences attributable to the ubiquitous traffic-related and other sources of air pollution in the urban environment of the cohort studied [2]. Several studies have documented increased prevalence of asthma by indoor air pollution using unprocessed biomass fuels [31]. The common source of indoor smoke to which children are frequently exposed in Arabian countries is the Arabian incense (bakhour) [32]. Incense burning produces continuous smoke, generating pollutants, such as toxic gases and chemical particles, including polycyclic aromatic hydrocarbons, carbon monoxide, benzene, and isoprene that easily accumulate indoors, especially under inadequate ventilation [33]. Exposure to incense smoke has been associated with several conditions, including respiratory symptoms, asthma, elevated cord blood IgE levels, contact dermatitis and cancer [32]. Taken together, these observations support the hypothesis that circulating PAHs concentration may influence the blood cytokine expression. To test this hypothesis *in vivo*, rats were given i.p. injections of benzopyrene for three weeks. Notably, this resulted in a significantly higher IL-4 mRNA expression in liver, lungs and blood tissues. However, there were no significant changes in expression of ACSL3 and IFN-γ mRNA expression in the above tissues except the lungs, in which IFN-γ mRNA was significantly over expressed (data not shown). Interestingly, this data in blood of animal model was in line with the human data. Benzo(a)pyrene (BaP) is one of the best qualified environmental PAHs, which along with several BaP-quinones (BPQs) are known to enhance IgE-mediated histamine release and cytokine production by altering specific signaling pathways. Pre-incubation of human basophils with BaP, 1,6-BPQ, and 3,6-BPQ, enhances histamine release and IL-4 production after short exposure time [34].

## Conclusions

In conclusion, the expression of cytokines we studied reflected the clinical status of the participants. The expression of IL-4 mRNA in PBMC was correlated with disease, and provided further evidence supporting the close relation of this cytokine with asthmatic condition. It does look that PAH level were raised in children with asthma and our animal study further corroborate the effect of PAH on IL-4 production which at least partly mediate the inflammatory response noticed in asthma. IL-4 augmentation observed here might be caused by continuous exposure to environmental PAH.

## Competing interests

The authors declare that they have no competing interests.

## Authors’ contributions

NMA and MSA conceived the study. SHA, HA and AKM carried out data acquisition and interpretation. AKM, SHA and KA analyzed the data and prepared the manuscript. MSC provided intellectual input and helped in drafting the final manuscript. All authors provided intellectual contributions to the manuscript and have read and approved the final version.

## Pre-publication history

The pre-publication history for this paper can be accessed here:

http://www.biomedcentral.com/1471-2431/14/17/prepub
